# Effects of different types and doses of whey protein on the physiological and intestinal flora in D-galactose induced aging mice

**DOI:** 10.1371/journal.pone.0248329

**Published:** 2021-04-15

**Authors:** Zuolin Ma, Fumei Zhang, Hongxin Ma, Xinghao Chen, Jiaqing Yang, Yiyan Yang, Xueying Yang, Xiaojing Tian, Qunli Yu, Zhongren Ma, Xueyan Zhou

**Affiliations:** 1 College of Food Science and Engineering, Gansu Agricultural University, Lanzhou, Gansu, China; 2 China-Malaysia National Joint lab, Biomedical Research Center, Northwest Minzu University, Lanzhou, Gansu, China; 3 Gannan Research Institute of Yak Milk, Hezuo, Gansu, China; 4 Department of Medicine, Northwest Minzu University, Lanzhou, Gansu, China; 5 College of Life Science and Engineering, Northwest Minzu University, Lanzhou, Gansu, China; University of Maine, UNITED STATES

## Abstract

The elderly usually suffer from many diseases. Improving the quality of life of the elderly is an urgent social issue. In this present study, D-galactose treated aging mice models were used to reveal the effects of different animal sources and different doses of whey protein (WP) on the immune indexes organs and intestinal flora. A total of 9 groups were set up, including normal control (NC), negative control (NS), positive control (Vc), low-, medium- and high-doses of cow WP intervention groups (CL, CM and CH for short, correspondingly) and low-, medium- and high-doses of goat WP intervention groups (GL, GM and GH for short, correspondingly). The body weight gain, thymus/body weight ratio, superoxide dismutase (SOD) activity, malondialdehyde (MDA) content, spleen immunoglobulins G (IgG), spleen interleukin-2 (IL-2) and spleen interleukin-2 (IL-6) were measured. Then, the intestinal contents were collected, and 16s genes of intestinal bacteria were sequenced to reveal the changes in bacterial flora structure. WP intervention significantly increased the weight gain, thymus/body ratio and SOD activity, but decrease the content of MDA. WP intervention increased some immune indicators. All the WP treated aging mice showed similar values of physiological indexes to that of the Vc group, even better. The relative abundance of *Lactobacillus* and *Stenotrophomonas* was increased and decreased, respectively, by both cow and goat WP. *Lactobacillus* may be involved in regulating the functional repair of organisms. In contrast, *Stenotrophomonas* might play a negative role in the immune and antioxidant capacity of the body. Combining physiological indicators and intestinal flora structure, low-concentration WP for cow and goat might be optimal for aging models.

## Introduction

Old people usually suffer from many diseases, such as hearing loss, immunosenescence intestinal diseases, metabolic diseases, cardiovascular and cerebrovascular diseases, etc. [1]. In order to improve the physical fitness of the elderly, some additional nutritional supplements are necessary. Protein supplementation is a good choice for old peoples [2,3]. However, there was little knowledge about the effects of different protein sources and concentrations on the body of the elderly. Previous studies have shown that dietary supplementation with milk serum protein can significantly reduce blood glucose, especially after meals, and is considered an adjunct strategy for the prevention and treatment of obesity and metabolic syndrome-related diseases in humans and animals [4–7]. Whey protein (WP) contains high doses of branched-chain amino acids (BCAAs), such as leucine (Leu), isoleucine (Ile), valine (Val), etc., which are important factors for muscle growth, development and repair in *vivo* [4,8]. WP is beneficial to mineral absorption, promotes protein synthesis, is sensitive to hormones, and reduces blood glucose and lipid levels in metabolic syndrome [5,7,9–13]. Cronin et al. found that WP supplementation experienced a significant alteration in the β-diversity of the intestinal flora [14]. Chen et al. reported that WP-derived early EGPs could increase the survival rate of aged male NOD mice, and decrease metabolic syndrome in immune infiltration [15]. These pieces of evidence indicated that WP could maintain body health and repair damage in many ways. WP has essential effects on intestinal microorganisms, but we know little about it in the elderly, not to mention the effects of WP from different animal sources on the elderly. It is unknown whether whey protein from different animal sources and concentrations has a positive effect on the elderly. In a 2014 study, the European Food Safety Authority noted several differences specifically between the whey proteins in cow and goat and concluded that three of the main whey proteins, alpha-lactalbumin, beta-lactoglobulin and serum albumin, were more highly concentrated in goat’s milk. The largest difference was in the serum albumin, which was significantly higher in goat’s milk, approximately three times as high as in cow’s milk at 1.2 versus 0.4 grams per liter [16].

Ageing can have drastic effects on the functions of the digestive system [17] and intestinal microbes also play a key role. The microbial community in the gut can be roughly divided into three categories according to the relationship with the body: beneficial bacteria, harmful bacteria and potential pathogenic bacteria [18]. The intestinal flora plays an extremely important role in human nutrition metabolism, development, immunity, and disease occurrence. It is also known as the "super tissue" or "virtual organ" of the human body [19,20]. The increased relative abundance of beneficial bacteria is beneficial to human health; on the contrary, if the harmful bacteria are dominant, the probability of causing related diseases will be significantly increased. The structure and stability of intestinal flora in different populations and development stages are different. In recent years, the intervention of intestinal flora has been widely used in the intestinal regulation of some special populations [18].

In oder to explore whether whey protein from different animal sources and concentrations has a positive effect on the elderly, we prepared a variety of WP (different animal sources and doses) to treat the aging mice models. The changes in intestinal flora structure induced by WP were measured using next-generation sequencing analysis. We hypothesize that WP from different animals or different doses has different promoting effects on the physical fitness of aging models. This present study will provide new insight into the mechanism of health improvement brought by WP in aged mice.

## Materials and methods

### Preparation of WP and its antioxidant activity in vitro

Fresh cow (Holstein) and goat (Guanzhong) milk were sampled from a grazing ranch in Yuzhong County, Gansu Province, China. The fresh milk was centrifuged (Thermofisher, Cambridge, MA, USA) at 4°C and 5000r/min for 30min. After centrifugation, the milk is divided into two layers; the upper layer is the fat part, the lower layer is the skim milk part (whey and the milk granule). Then, adjusting the pH of skim milk (the lower layer) to 4.6 with 1 M HCl at room temperature (RT). Subsequently, the skim milk was water-bathed at 40°C for 20min, followed by centrifuging at 8000r/min for 20min (4°C). The supernatant (whey) was collected and freeze-dried. The cow whey protein (CWP) and goat whey protein (GWP) was stored in dry conditions at -4°C until used.

### Animal experiments

All animals used were reviewed and approved by Lanzhou University of China with the ethical review number of xbmu-sm-201902. All animals were housed, cared, and used in compliance with the Guide for the Care and Use of Laboratory Animals and housed and used in an Association for the Assessment and Accreditation of Laboratory Animal Care (AAALAC) Program. A total of 60 healthy adult female Kunming White mice strain (6 weeks old, 25–30g body weight) were provided by the Experimental Animal Center of Institute of Genetics and Developmental Biology at the Chinese Academy of Sciences. The aging model was induced by subcutaneous injection with 10% D-galactose dissolved in normal saline (0.25mL/20g/day) for 6 weeks. A total of 9 groups were designed, including normal control group (NC), Negative control (NS), Positive control (Vc), low-, medium- and high-doses of cow WP intervention groups (CL, CM and CH for short, correspondingly) and low-, medium- and high-doses of goat WP intervention groups (GL, GM and GH for short, correspondingly). We selected 45 aging models with similar body weight, and then randomly divide them into 9 groups (5 individuals in each group). Except for the NS group, the other groups continued to be injected with 10% D-galactose dissolved in normal saline (0.25mL/20g/day) by intraperitoneal injection. Meanwhile, different types and different doses of WP were given to the aging models for 7 weeks (intragastric administration). NC mice were given an equivalent weight of placebo (saline, intragastric administration). The processing details are shown in **Table 1**.

**Table 1:** Details of different treatment in different groups.

Groups	Treatment	
	Intraperitoneal injection	Intragastric administration
NC	--	Ns 0.5mL/mouse/d
NS	10% D-galactose 0.25mL/20g/d	Ns 0.5mL/mouse/d
Vc	10% D-galactose 0.25mL/20g/d	Vc 300mg/kg/d
CL	10% D-galactose 0.25mL/20g/d	CWP 100mg/kg/d
CM	10% D-galactose 0.25mL/20g/d	CWP 200mg/kg/d
CH	10% D-galactose 0.25mL/20g/d	CWP 400mg/kg/d
GL	10% D-galactose 0.25mL/20g/d	GWP 100mg/kg/d
GM	10% D-galactose 0.25mL/20g/d	GWP 200mg/kg/d
GH	10% D-galactose 0.25mL/20g/d	GWP 400mg/kg/d

CWP: Cow whey protein; GWP: Goat whey protein; NC: Normal control; NS: Negative control, normal saline treatment; Vc: Positive control, vitamin C treatment; CL, CM, CH represents low-, middle-, high-concentration cow whey protein intervention group, respectively. GL, GM, GH represents low-, middle-, high-concentration goat whey protein intervention group, respectively.

After 7 weeks of WP intervention, the superoxide dismutase (SOD) activity and malondialdehyde (MDA) content in serum were measured with 5 biological replicates to justify whether the aging model is successful [21]. The SOD activity and MDA content in serum were measured using the SOD and MDA activity test kit provided by Nanjing Jiangcheng Bioengineering Institute. Besides, the body and thymus weights were measured with 5 biological replicates to assess animal immune indexes. Then, three mice were randomly selected to collect intestinal tracts for the detection of intestinal bacteria by Next-generation Sequencing (NGS) of the 16S rRNA gene amplicons method.

### Spleen immunoglobulins G (IgG) and cytokine detection

For the preparation of spleen extract, firstly, we weigh 100 mg of the spleen tissue sample. Then, adding 10 times the volume of 1X Phosphate buffered saline (PBS) containing 1% protease inhibitor and 1% phosphatase inhibitor to the sample, quickly freezing in liquid nitrogen, and grinding for 4 minutes with a grinder. Next, the ground tissue was centrifuged at 12000r/min at 4°C for 10 minutes, and the supernatant was taken and stored at -20°C. Immunoglobulins G (IgG) was measured using the Mouse immunoglobulin G ELISA Kit (Huamei, Wuhan, China). The levels of spleen interleukin-2 (IL-2) and interleukin-2 (IL-6) in the spleen extract were measured by ELISA (Mouse IL-2 ELISA Kit and Mouse IL-6 ELISA Kit for IL-2 and IL-6 measuring; Lianke, Hangzhou, China). ELISAs were performed according to the manufacturer’s instructions.

### Intestinal microbial DNA isolation and 16sRNA sequencing

Total DNA samples were extracted from intestinal contents using MoBioPowersoil DNA extraction kits (MoBio, Carlsbad, CA, USA) following the manufacturer’s instructions. The DNA quality was determined using 1% agarose gel electrophoresis. DNA quantification was performed using a Qubit2.0 DNA Assay Kit (Sangon Biotech Co., Ltd, Shanghai, China). High-quality DNA was selected for subsequent sequence library construction and sequencing. Amplification of the V3 and V4 region of the 16S rRNA gene were performed base on the DNA templates conducted using the 515 (5’-GTGCCAGCMGCCGCGGTAA-3’) and 806 (5’-GGACTACHVGGGTWTCTAAT-3’) primers. The PCR amplifications were carried out in an Eppendorf master cycler in a 50 μL reaction system, which contains 10 ng of genomic DNA, 0.5 μL dNTP (10 mM each), 0.5 μL of each PCR primer (50 uM) and 0.5 μL of Taq (5 U/uL). The PCR protocol was as follows: an initial denaturation 10 min at 95°C, followed by 30 cycles of 95°C for 15 s, annealing at 60°C for 15 s and extension at 72°C for 30 s; final extension at 72°C for 5 min. Three technical duplicates with negative controls for each gene were conducted at the same conditions. GeneJET RNA Purification Kit (Thermofisher, Cambridge, MA, USA) was used to collect the target fragments of DNA. The densities of the collected fragments were detected by Qubit2.0 (Invitrogen Life Tech., Carlsbad, MA, USA), and quality control was performed with Agilent 2100 Bioanalyzer (Agilent, Pal Alto, CA, USA). Quantitative PCR (qPCR) was performed to test the efficiency of the adapters. Then, the libraries were diluted to a proper concentration for sequencing. IonS5TMXL system (Thermofisher, Cambridge, MA, USA) was used to accomplish the sequencing under Single-End (SE) 600bp mode.

### Sequence data analysis

Raw reads from different samples were separated according to the barcode adapter and PCR primer sequences. Data quality filtering was carried out by Cutadapt (v1.9.1, https://cutadapt.readthedocs.io/en/stable/) quality-controlled process under specific filtering conditions. Reads were submitted to the Silva database (https://www.arb-silva.de/) for chimera sequences identification via the UCHIME algorithm (v4.1, https://drive5.com/usearch/manual/uchime_algo.html), then all the detected chimera sequences were filtered out. Operational taxonomic units (OTUs) clustering was conducted using Uparse software (v7.0.1001; http://drive5.com/uparse/) based on the threshold of 97% identity. The abundance (reads number) of OTUs in each sample was calculated, and OTUs with more than two reads were used for further analysis. Alpha diversity indicators, including Chao1, ACE, observed OTUs, Shannon, and Simpson, were calculated by QIIME (v1.8.0, http://qiime.org/) software and displayed with R software (v2.15.3, https://www.r-project.org/). Meanwhile, Beta diversity on both weighted and unweighted unifrac was calculated by QIIME software (v1.7.0, http://qiime.org/). Principal Co-ordinates Analysis (PCoA) of samples was performed based on the Unweighted UniFrac distance of the beta diversity index. SILVA rRNA database (http://www.arb-silva.de/) on Mothur website (http://www.mothur.org/wiki/RDP_reference_files) was queried for the annotation of the OTUs. OTUs relative abundances (from phylum to species level) were calculated, and taxonomy assignment (phylum ~ species level) was performed using the Ribosomal Database Project (RDP) classifier (80% confidence). Function prediction analysis was achieved by the nearest neighbor method based on the minimum 16S rRNA sequence similarity by extracting the KEGG database prokaryotic whole-genome 16S rRNA gene sequence via the Tax4Fun R package. All the analysis was achieved through our in-house scripts.

### Statistical analysis

Data were expressed as the mean ± standard deviation (SD). The SPSS 22.0 (https://www.ibm.com/support/pages/spss-statistics-220-available-download) software was employed for the statistical analysis. All experimental data were expressed as mean ± SD, and differences between groups or treatments were analyzed using the unpaired t-test. Differences across tissues were analyzed using one-way ANOVA test. *P* < 0.05 was considered a significant difference. The results were displayed by Prism 8 (https://www.graphpad.com/scientific-software/prism/) software.

## Results

### Effects of WP intervention on aging mice models

After 7 weeks of different treatments, the mice showed significant differences. The SOD and MDA in C and Vc was significantly higher and lower than that in NS, respectively; the SOD and MDA in Vc was similar with that in C, indicating that the aging model is successful. Of all these 9 groups, the mice in the NS group had the lowest value of weight gain, thymus/body ratio, and SOD activity but the highest value of MDA content (**Table 2**).

**Table 2:** Effects of whey protein intervention on physiological indexes of aging mice.

Groups	Weight gain (%)	Thymus/body (mg/g)	SOD activity (U/mL)	MDA content (nmol/mL)
C	17.626±2.909a	2.967±0.2082a	82.444±2.296a	2.0000±0.54358a
NS	0.356±0.311c	2.367±0.1155b	42.500±1.516b	5.7632±0.68959b
Vc	3.516±1.576b	2.967±0.2517a	76.125±4.788a	3.0514±0.96282a

NC: Normal control; NS: Negative control, normal saline treatment; Vc: Positive control, vitamin C treatment; Different letters indicate different statistical significances (*p*<0.05). SOD: Superoxide dismutase; MDA: Malondialdehyde.

**Fig 1:**
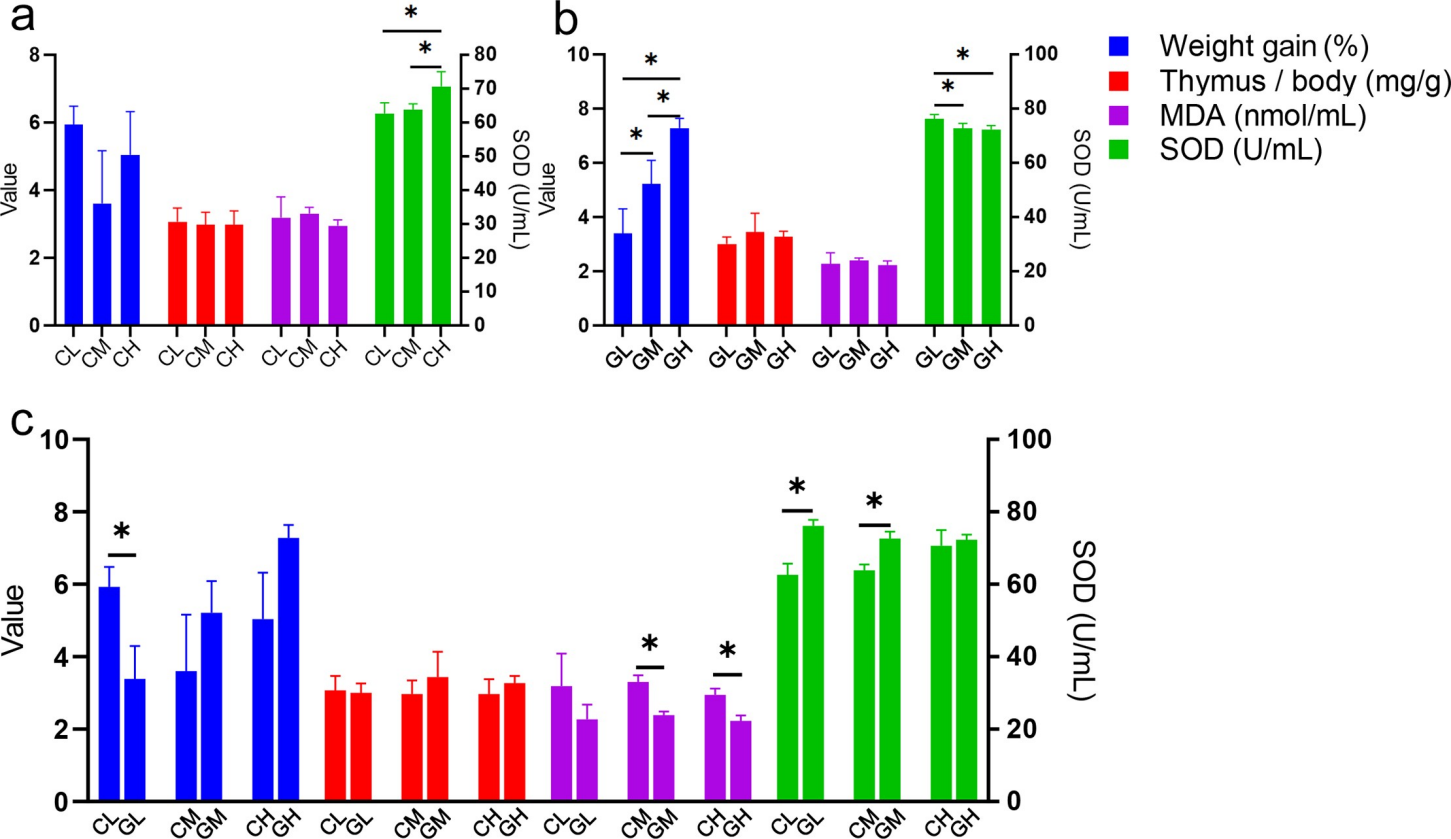
The physiological indexes among different whey protein treated groups. a. physiological indexes in cow whey protein treated groups; b. physiological indexes in goat whey protein treated groups; c. comparisons of different types of whey proteins at the same concentration. * Correlation is significant at the 0.05 level. The left-hand ordinate showed the value of weight gain (%), Thymus/body (mg/g) and MDA content (nmol/mL); The right-hand ordinate showed the SOD activity (U/mL). NC: Normal control; NS: Negative control; Vc: Positive control; CL, CM, CH represents low-, middle-, high-concentration cow whey protein intervention group, respectively. GL, GM, GH represents low-, middle-, high-concentration goat whey protein intervention group, respectively.

All the WP treated aging mice showed similar values of physiological indexes to that of the Vc group, even better. For CWP treated groups, CH had higher SOD activity than CM and CL (**Fig 1A**). For GWP treated groups, the weight gain in GH was significantly higher than that in GM and GL. The SOD activity value in GL was significantly higher than that in GM and GH (**Fig 1B**). WP from different animal sources showed different physiological values at the same concentration: CL had a higher weight gain than GL; CM and CH had higher MDA content than GM and GH, respectively; CL and CM showed lower SOD activity than GL and GM, respectively (**Fig 1C**).

### Effects of WP intervention on serum antibody and cytokine

**Fig 2:**
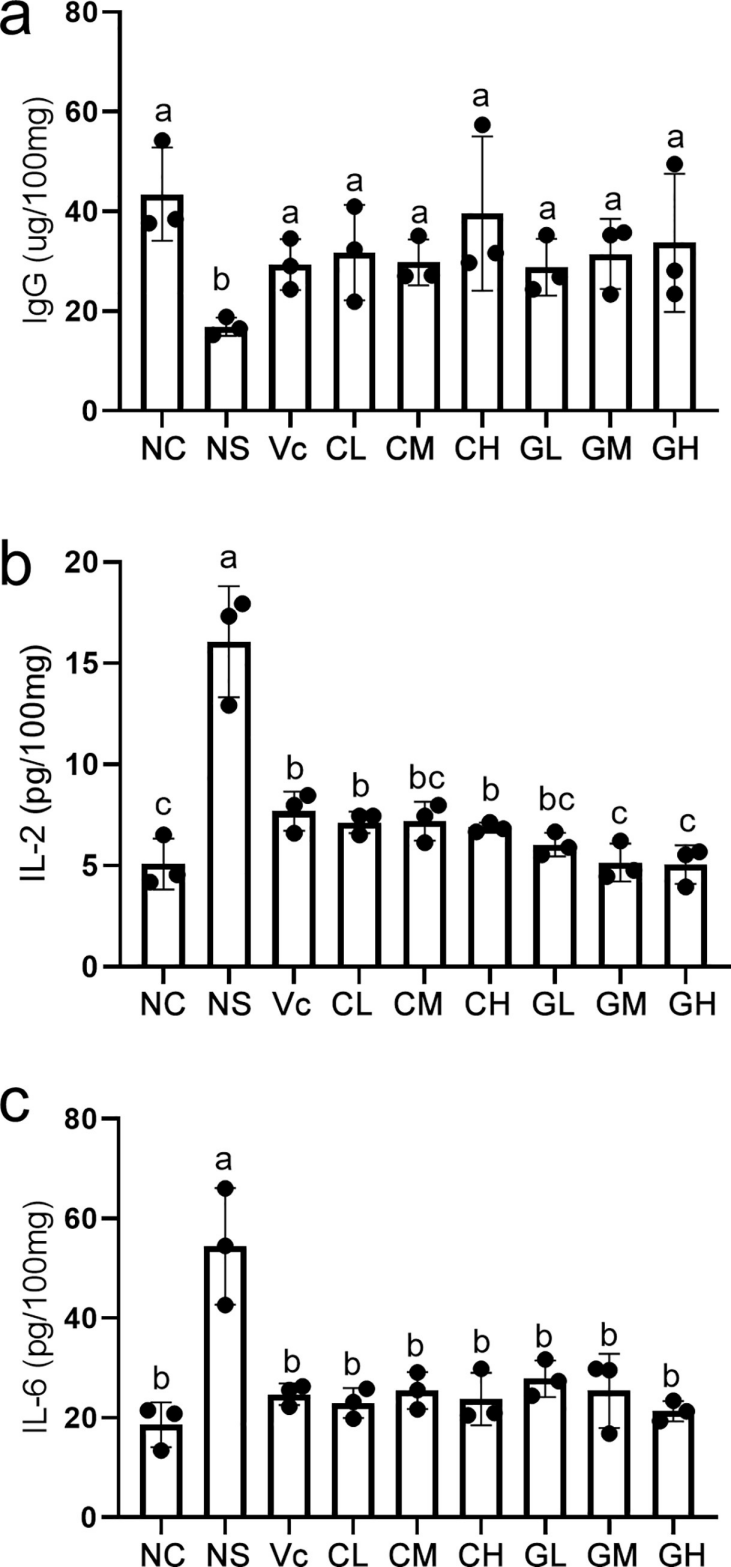
The immunity indexes among different whey protein treated groups. a, b and c showed the content of immunoglobulins G (IgG), interleukin-2 (IL-2) and interleukin-2 (IL-6) in spleen. Each black dot represents a biological repeat. Different letters indicate different statistical significances (p<0.05). NC: Normal control; NS: Negative control; Vc: Positive control; CL, CM, CH represents low-, middle-, high-concentration cow whey protein intervention group, respectively. GL, GM, GH represents low-, middle-, high-concentration goat whey protein intervention group, respectively.

By measuring the contents of IgG, IL-2 and IL-6, we found that the immune indicators of mice after intervention with WP are significantly different from that of negative control. The concentration of IgG in NS was significantly lower than that in C and Vc groups. But after intervention with WP, the concentration of IgG increased significantly in all the treated mice, which were significantly higher than that in NS group (**Fig 2A**). In addition, the IL-6 and IL-2 showed similar trends. Ns had the highest concentration of IL-2 and IL-6, which was significantly higher than the other groups (**Fig 2B and 2C**), indicating that WP intervention significantly decreased the IL-2 and IL-6 levels in the aging model.

### Sequence data summary

A total of 2.2Mb sequence reads were obtained from these 27 samples. After trimming the sequencing adapter and filtering out the low-quality reads, we finally identified 2.1 Mb reads with 77,710 reads per sample. The reads quality and database were listed in **S1 Table**. In addition, the rarefaction curves (**S1 Fig**) and species accumulation curves (**S2 Fig**) showed that the increasing of OTUs tended to be flat with the increase of sequencing reads numbers and samples, respectively, which indicated the amount of data and samples number were enough. Besides, the Principal Components Analysis (PCA) and phylogenetic analysis results revealed the excellent repeatability of all the test samples (**S3 Fig**). All the basic statistical results indicated the excellent sequence data quality and consistency of repetitions. All raw reads were submitted to the BIGSUB GSA database (https://bigd.big.ac.cn/gsub/) under the accession number CRA003438.

### Effects of different concentration of WP on intestinal flora diversity

Different doses of WP showed a certain impact on the α-diversity of intestinal flora, but not widely (**Table 3**).

**Table 3:** Alpha indexes in different groups.

	Observed_species	Shannon	Simpson	Chao1	ACE	PD_whole_tree
C	249.31±23.21 ^ab^	3.51±0.11 ^b^	0.81±0.01 ^ab^	299.71±33.31 ^ab^	318.41±38.91 ^ac^	25.21±3.31 ^ab^
NS	147.01±24.11 ^b^	2.81±0.61 ^c^	0.71±0.11 ^b^	162.21±28.81 ^b^	172.61±32.21 ^b^	33.61±28.21 ^ab^
Vc	268.71±55.91 ^a^	3.21±0.31 ^bc^	0.81±0.01 ^b^	309.91±79.71 ^a^	323.41±82.91 ^a^	25.31±7.11 ^ab^
CL	199.71±46.21 ^ab^	3.31±0.31 ^bc^	0.81±0.01 ^ab^	227.31±53.11 ^ab^	236.31±48.31 ^abc^	34.91±6.81 ^ab^
CM	185.01±69.61 ^ab^	3.11±0.11 ^bc^	0.81±0.01 ^b^	199.81±77.11 ^ab^	205.91±83.51 ^abc^	20.81±5.21 ^b^
CH	232.31±52.81 ^ab^	4.31±0.41 ^a^	0.91±0.01 ^a^	249.51±55.91 ^ab^	257.91±53.31 ^abc^	55.91±20.71 ^ab^
GL	145.71±38.91 ^b^	3.01±0.31 ^bc^	0.81±0.01 ^b^	163.11±42.01 ^b^	176.01±46.41 ^bc^	41.81±25.21 ^ab^
GM	270.31±70.31 ^a^	3.41±0.41 ^bc^	0.81±0.11 ^ab^	311.51±89.21 ^a^	309.71±81.71 ^abc^	66.51±46.61 ^ab^
GH	219.31±84.41 ^ab^	3.51±0.41 ^b^	0.81±0.01 ^ab^	253.51±107.41 ^ab^	257.81±110.71 ^abc^	70.81±27.71 ^a^

NC: Normal control; NS: Negative control, normal saline treatment; Vc: Positive control, vitamin C treatment; CL, CM, CH represents low-, middle-, high-concentration cow intervention group, respectively. GL, GM, GH represents low-, middle-, high-concentration GWP intervention group, respectively.

**Fig 3:**
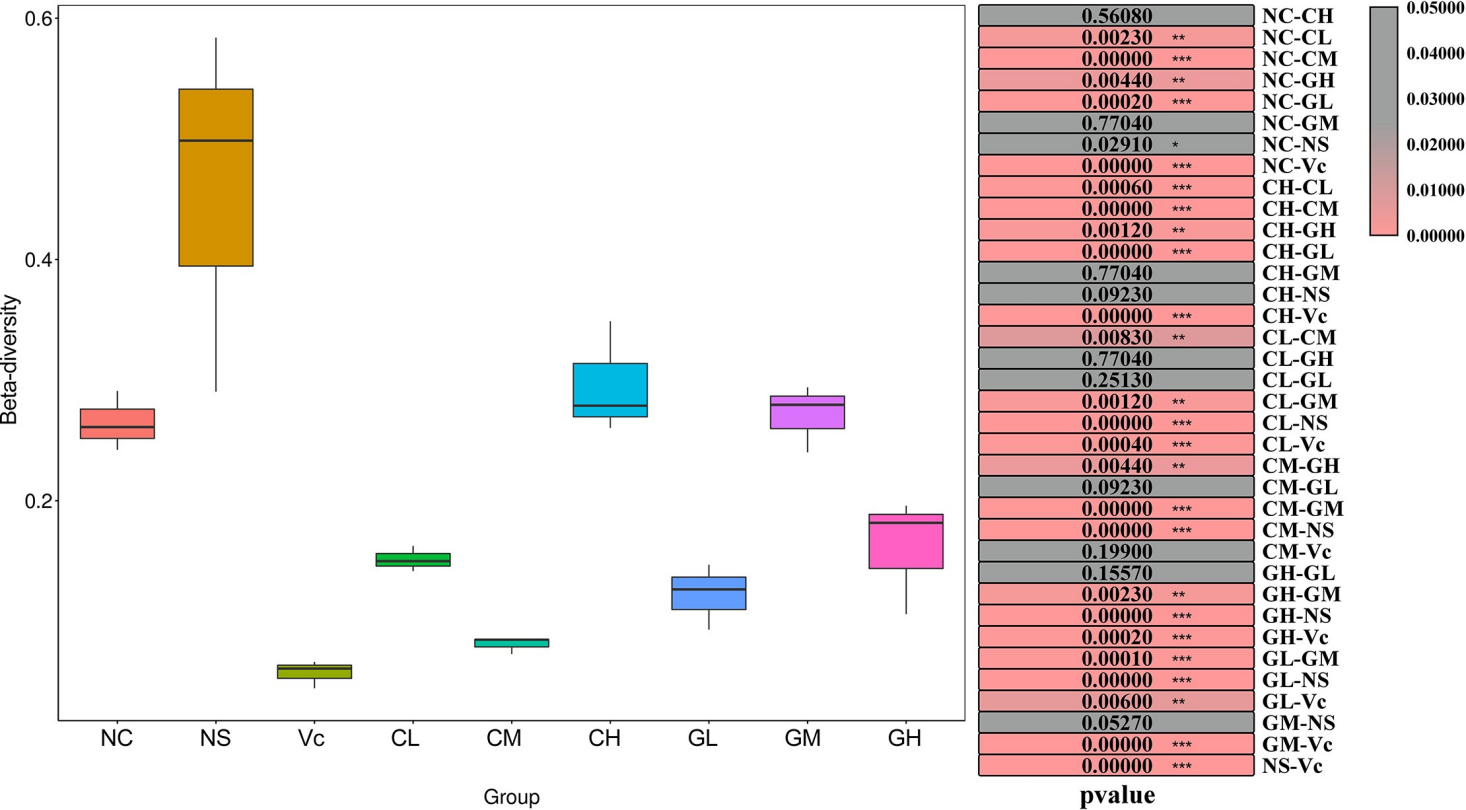
β-diversity of different groups. The different colored boxes on the left represent different groups. The right side of the figure shows the p-value between two comparisons. The β-diversity was calculated by Wilcox test. NC: Normal control; NS: Negative control; Vc: Positive control; CL, CM, CH represents low-, middle-, high-concentration cow whey protein intervention group, respectively. GL, GM, GH represents low-, middle-, high-concentration goat whey protein intervention group, respectively.

As expected, all α—indexes values in group NS were the lowest or the second-lowest of all groups. For WP treated groups, GM had the highest observed_species and chao1 value, which was similar to that of the Vc group. The higher observed_species and chao1 value indicated that there were more species of intestinal bacteria in the GM group. GL had similar observed_species and chao1 values with the NS group, indicating the fewer species of intestinal bacteria. The Shannon value in CH was significantly higher than that in other groups. Similarly, the value of the Simpson index in CH was the highest of all groups. For β-diversity analysis, NS was significantly higher than NC, Vc, CL, CM, GL and GH. β-diversity value in Vc was the lowest in all groups. The WP treated groups of different animal sources and different doses showed different levels of β-diversity (**Fig 3**).

### Effects of WP on bacteria community

**Fig 4:**
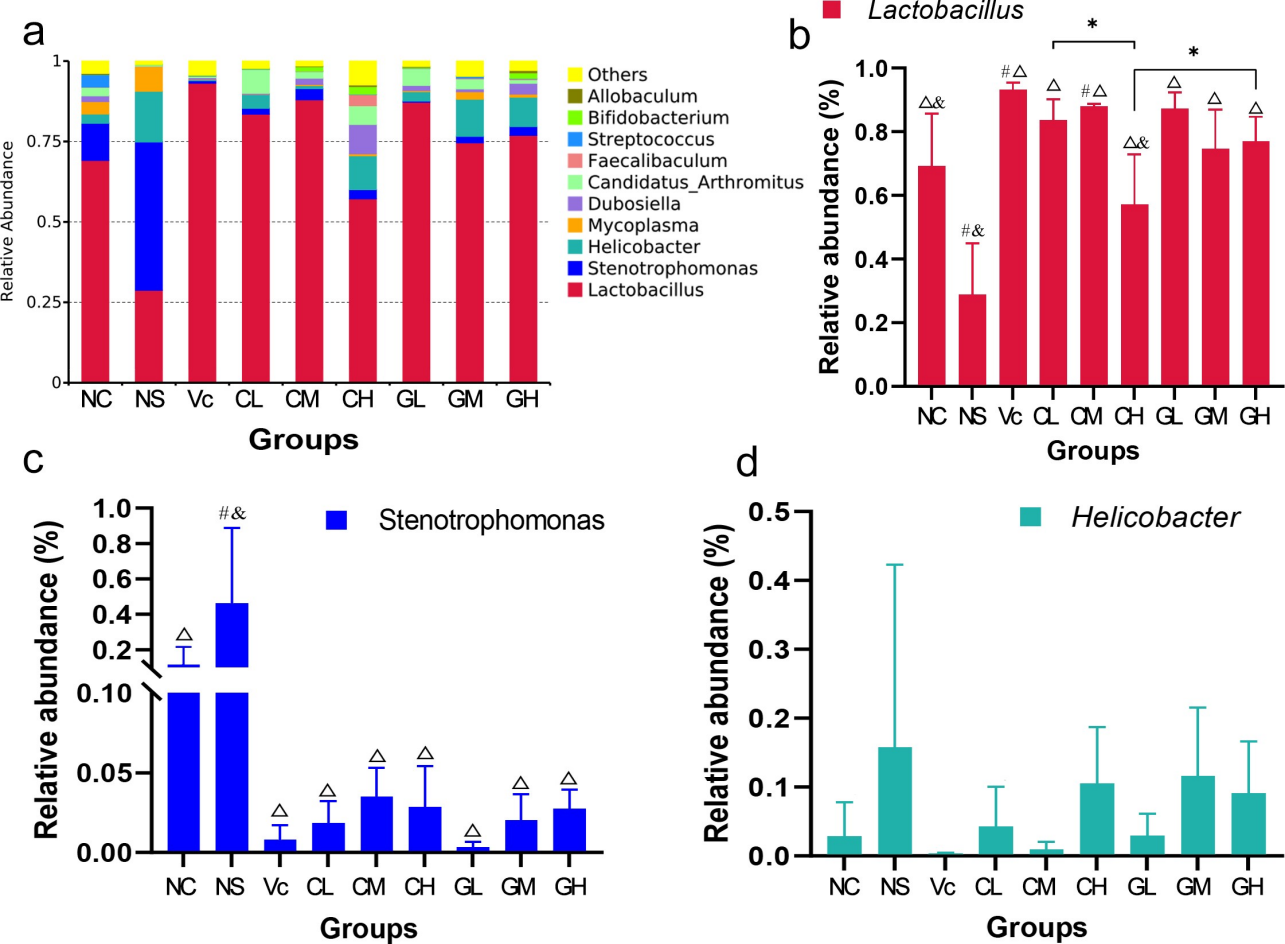
Results of relative abundance of intestinal bacteria. a. the relative abundance of intestinal bacteria in all groups; b, c and d. statistical results of relative abundance of *Lactobacillus*, *Stenotrophomonas*, and *Helicobacter* in different groups, respectively. NC: Normal control; NS: Negative control; Vc: Positive control; CL, CM, CH represents low-, middle-, high-concentration cow whey protein intervention group, respectively. GL, GM, GH represents low-, middle-, high-concentration goat whey protein intervention group, respectively; #, *p* < 0.05, compared with the C; ρ, *p* < 0.05, compared with the NS; &, *p* < 0.05, compared with the Vc; * Correlation is significant at the 0.05 level.

Overall, *Lactobacillus*, *Stenotrophomonas*, and *Helicobacter* were the dominant bacteria in all groups. As expected, the dominant bacteria in Vc were quite different from that of NS. The abundance of dominant bacteria in non-treated control (C) was intermediated between Vc and NS (**Fig 4A**). The results showed that different WP treatments had different effects on aging models. *Lactobacillu* was the most abundant bacteria in group C, Vc, and WP treated groups, which was significantly higher than that of NS (**Fig 4B**). For the same type of WP, different doses had significant effects on the abundances of *Lactobacillu*. The abundance of *Lactobacillu* in CL was significantly higher than that of CH. At the same concentration, there were also differences between groups treated with CWP and GWP, such as the abundance of *Lactobacillu* in GH was significantly higher than that in CH (**Fig 4B**). For *Stenotrophomonas* (the second-highest abundance), the relative abundance of which in NS was significantly higher than other groups (**Fig 4C**). No significant difference found in the relative abundance of *Helicobacter* (the third-highest abundance) among all these groups (**Fig 4D**). Interestingly, Vc and WP could effectively suppress the abundance of *Mycoplasma*. Among all groups, *Mycoplasma* was the most abundant in the NS group, followed by the C group. However, with the supplement of Vc and WP, the relative abundance of *Mycoplasma* decreased dramatically.

### Correlation analysis results

**Fig 5:**
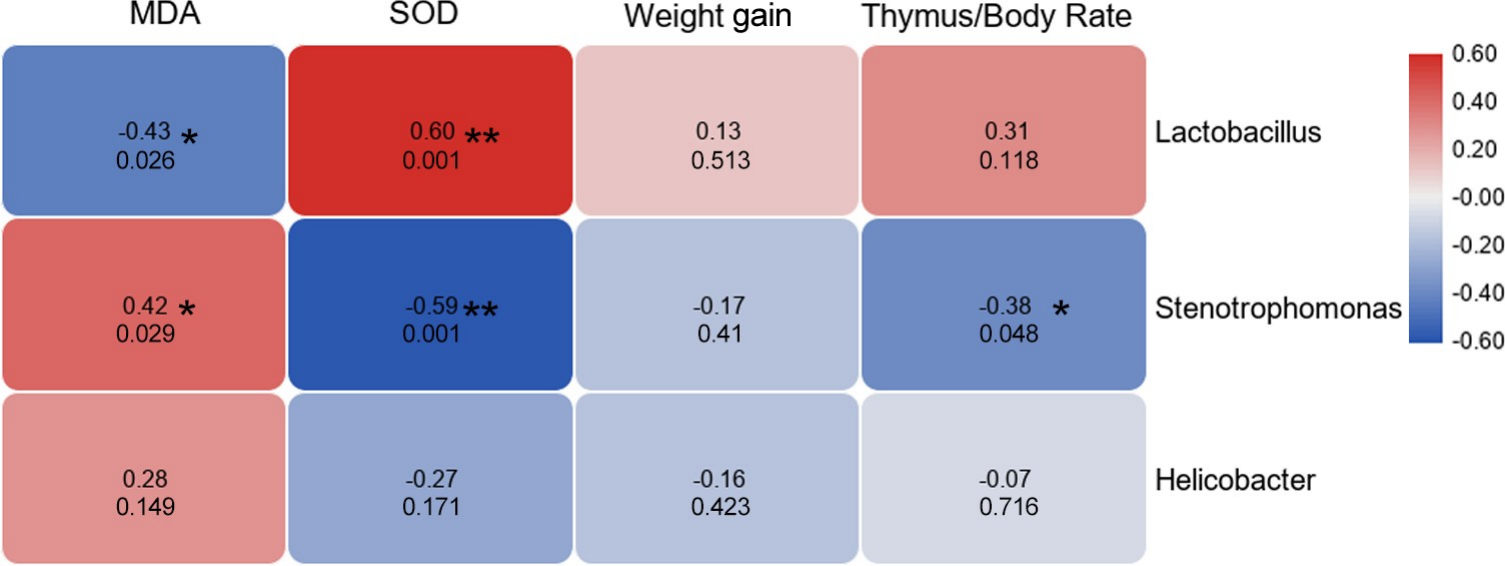
Correlation analysis results. Correlation analysis results among the abundance of dominant bacteria and physiological indicators. The redder the color, the stronger the positive correlation, and the bluer the color, the stronger the negative correlation. The numbers in cells indicate the correlation coefficient (upper) and p value (bottom). SOD: Superoxide dismutase; MDA: Malondialdehyde.

In order to study the correlation between different indicators and physiological data, Pearson analysis was performed. Correlation analysis results among the abundance of dominant bacteria and physiological indicators, we found that *Lactobacillus* was negatively and positively correlated with MDA and SOD, respectively. The relative abundance of *Stenotrophomonas* was positively correlated with MDA content but negatively correlated with SOD and thymus/body ratio. Moreover, the abundance of *Helicobacter* showed no correlation with the physiological data (**Fig 5**).

### Functional prediction analysis results

**Fig 6:**
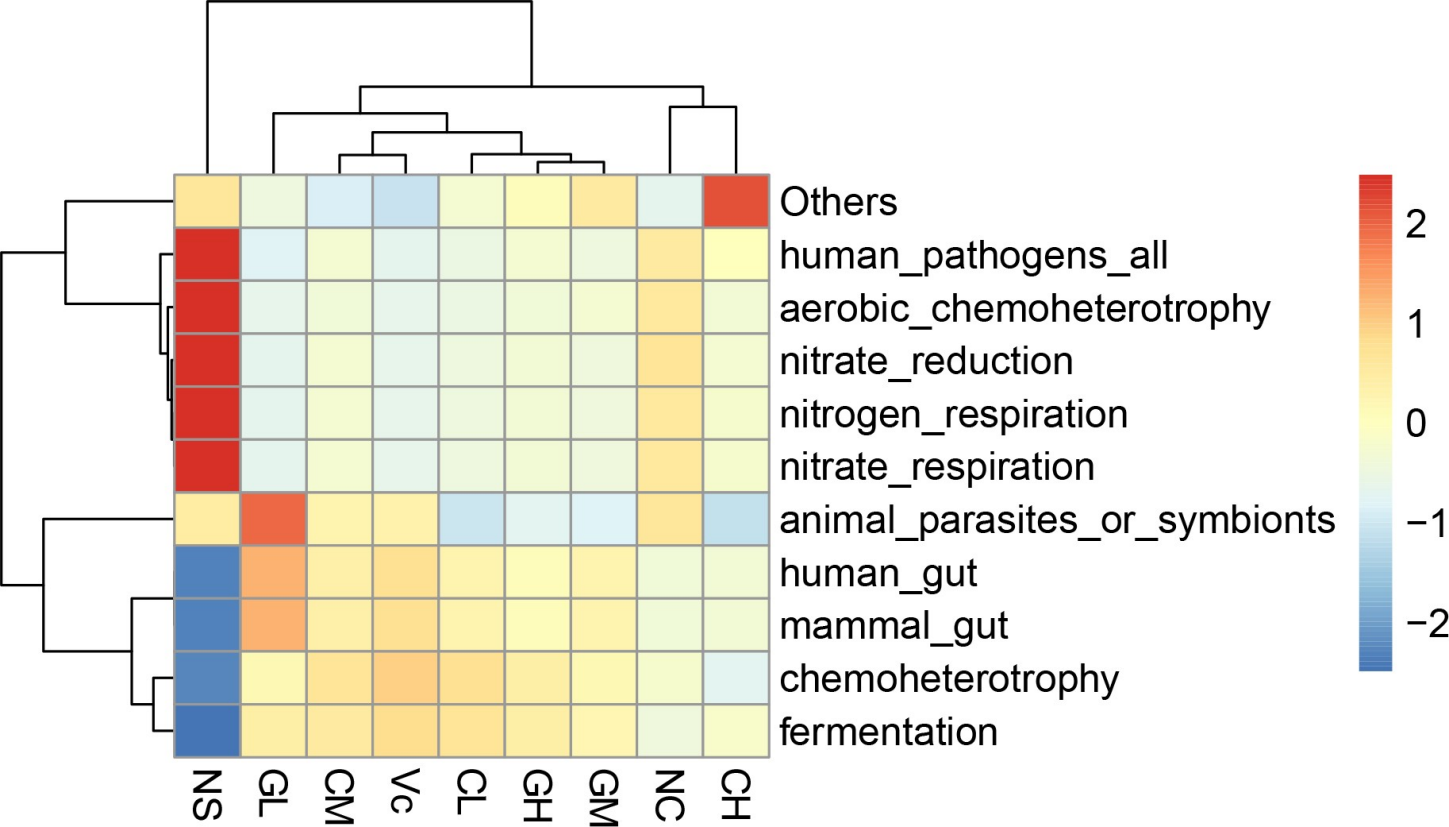
Functional prediction analysis results. Different colors represent the level of correlation between groups and functions. The redder the color, the stronger the correlation; the bluer the color, the weaker the correlation. NC: Normal control; NS: Negative control; Vc: Positive control; CL, CM, CH represents low-, middle-, high-concentration cow whey protein intervention group, respectively. GL, GM, GH represents low-, middle-, high-concentration goat whey protein intervention group, respectively.

Fermentation, chemoheterotrohy, mammal_gut, and human_gut functions of NC group mice were significantly reduced by D-galactose treatment, but the functions were restored after Vc and WP supplementation (**Fig 6**). In addition, the NC group showed stronger functions in nitrate respiration, nitrogen respiration, nitrate reduction, good oxidative chemotacticity, and human pathogens. However, these enriched functions mentioned above in WP treated groups were weaker. Also, a similar situation occurred in groups Vc and C.

## Discussion

Whey protein is known for its muscle accretion effects, which is often used as a commercial supplement in elite sport and amateur fitness milieu [22]. What’s more, WP had a positive influence on energy metabolism, even on appetite control [23–26]. WP and lactose were the most important components of milk, both of which can act as useful substrates for bacteria, and approximately 10% of protein intake reaches the human colon [27]. In the present study, we evaluated the effect of different types and doses of WP on aged mice intestinal microbiota.

In our study, the mice in group C gained more than 17 g of weight, while mice in the negative control (NS) gained almost no weight. This difference might be due to the decline in the digestive capacity of intestines in the aging models. However, the addition of WP significantly increased the body weight, even exceeding that of the Vc group, indicating the promoting effect on the body constitution in aged mice. MDA is a direct product of lipid peroxidation. It acts as a biomarker to the cell damage induced by oxidative stress [28]. Previous research reported that the erythrocyte MDA levels increased with age in healthy subjects [29]. What’s more, the lipid peroxidation products accumulate with age [30]. Our results showed that the WP treated mice decreased the MDA contents, indicating the increasing ability to cope with oxidative stress. Correspondingly, the increased SOD activity also showed the benefits of WP to the body. The thymus and spleen are important immune organs, which are the major players in maintaining immune homeostasis [31]. The organ weight of the thymus reflects the immune function and immune status of the body and the assessment of immunotoxicity [32]. The increased thymus/body ratio by WP indicated that the immunity of the aging mouse model might be improved. In addition, the content of IgG, IL-6 and IL-2 in the spleen also suggested that the immunity of the aging model might be improved after the intervention of WP. In addition, at the same concentration, CWP had higher weight gain and MDA content, but lower SOD activity than GWP. The α-diversity results showed that WP treatment could significantly increase the diversity of intestinal bacteria, indicating that WP can promote the rebuilding of gut microbes in aging model mice. Different WPs greatly affected the relative abundance of dominant bacteria, such as *Lactobacillus*, *Stenotrophomonas*, and *Helicobacter* genera. *Lactobacillus* is a common member of the small intestine of humans and other mammals [33,34]. *Lactobacillus* had a strong ability to metabolize carbohydrates and produce acids, and they could also synthesize dextran and heteropolysaccharide [35–38]. Previous studies had reported that some beneficial bacteria such as *Bacteroidetes*, *Lactobacillus*, and *Bifidobacteria* are reduced in the gut flora of the elderly [39–41]. We found that low doses of cow and goat WPs can significantly improve the relative abundance of *Lactobacillus*, which was close to the level of positive control (group Vc). The aged mice benefited from the increasing of *Lactobacillus*. Fooks et al reported that *Lactobacillus* plantarum and *B*. *bifidum* had potent inhibition of enteropathogen growth in vitro [42], which could keep the organism healthy, especially for the aged mammal. Interestingly, we found that higher doses of cow and goat WP decreased the mean value of the relative abundance of *Lactobacillus* (**Fig 2B**), indicating that a low concentration of cow and goat WP had positive effects on the relative abundance of *Lactobacillus*. Therefore, we speculate that the positive effect of low-concentration WP (form cow or goat) on the aging model may be more obvious. The relative abundance of *Stenotrophomonas* was also greatly influenced by WP treatment. We found that all the aged mice were infected with *Stenotrophomonas*, which had been associated with high morbidity and mortality in severely immunocompromised and debilitated individuals [43,44]. WP diet showed inhibitory effects on *Stenotrophomonas*, and there was no significant difference between WPs of different animals. So, we speculated that WP, regardless of type or concentration, can effectively inhibit the growth of *Stenotrophomonas* and have a positive effect on the treatment of *Stenotrophomonas* infection. Similarly, the cow and goat WP showed inhibitory effects on *Helicobacter* (not statistically significant). Fox et al. isolated a novel *Helicobacter* species (*Helicobacter bilis sp*. *nov*.) in aged mice [45], also, we found that the abundance of *Helicobacter* in NC was higher than other groups. *Helicobacter* species are able to thrive in the acidic mammalian stomach by producing large quantities of the enzyme urease, which locally raises the pH from about 2 to a more biocompatible range of 6 to 7 [46]. This indicated the beneficial role of WP in inhibiting harmful bacteria in the gut of aged mice.

Besides, we found that the abundance of *Lactobacillus* and *Stenotrophomonas* showed a significant correlation with physiological indexes (**Fig 3B**). This suggests that *Lactobacillus* may be involved in regulating the body’s anti-aging effects. In addition, the present data showed that the inhibition of *Stenotrophomonas* might play a positive role in improving the immune and antioxidant capacity of the body. The function prediction analysis results showed that whey supplement could increase intestinal fermentation function, and this is mainly achieved by *Lactobacillus* bacteria. Sousa et al. had reviewed that the branched-chain amino acids in WP were related to muscle growth, build, and repair [24], which was consistent with the prediction of intestinal flora function in our results.

## Conclusion

Whey protein treatment could increase the weight gain, thymus/body ratio, and SOD activity and decrease the content of MDA. Also, immune indicators were improved by WP intervention. All the whey protein treated aging mice had similar values of physiological indexes to that of the Vc group, even better. Combining with the physiological indicators and intestinal flora diversity, low doses of cow and goat whey protein might be optimal for aging models. Whey protein benefits intestinal health by increasing the relative abundance of beneficial bacteria (such as *Lactobacillus*) and inhibiting the growth of harmful bacteria (such as *Stenotrophomonas*). The effects of different types of whey protein on the gut health of aged mice are different. *Lactobacillus* may be involved in regulating functional repair of organisms. While *Stenotrophomonas* might play a negative role in the immune and antioxidant capacity of the body.

## Supporting information

S1 Fig Rarefaction curve of Observed_species. (TIF)

S2 Fig Species accumulation curve. (TIF)

S3 Fig PCA results of all the samples. (TIF)

S1 Table Sequence data summary. (XLSX)
