# Supplementary figures and images for: Effects of different types and doses of whey protein on the physiological and intestinal flora in D-galactose induced aging mice
Source: PLoS One. 2021 Apr 15;16(4):e0248329. doi: 10.1371/journal.pone.0248329 (PMC8049228; doi:10.1371/journal.pone.0248329)

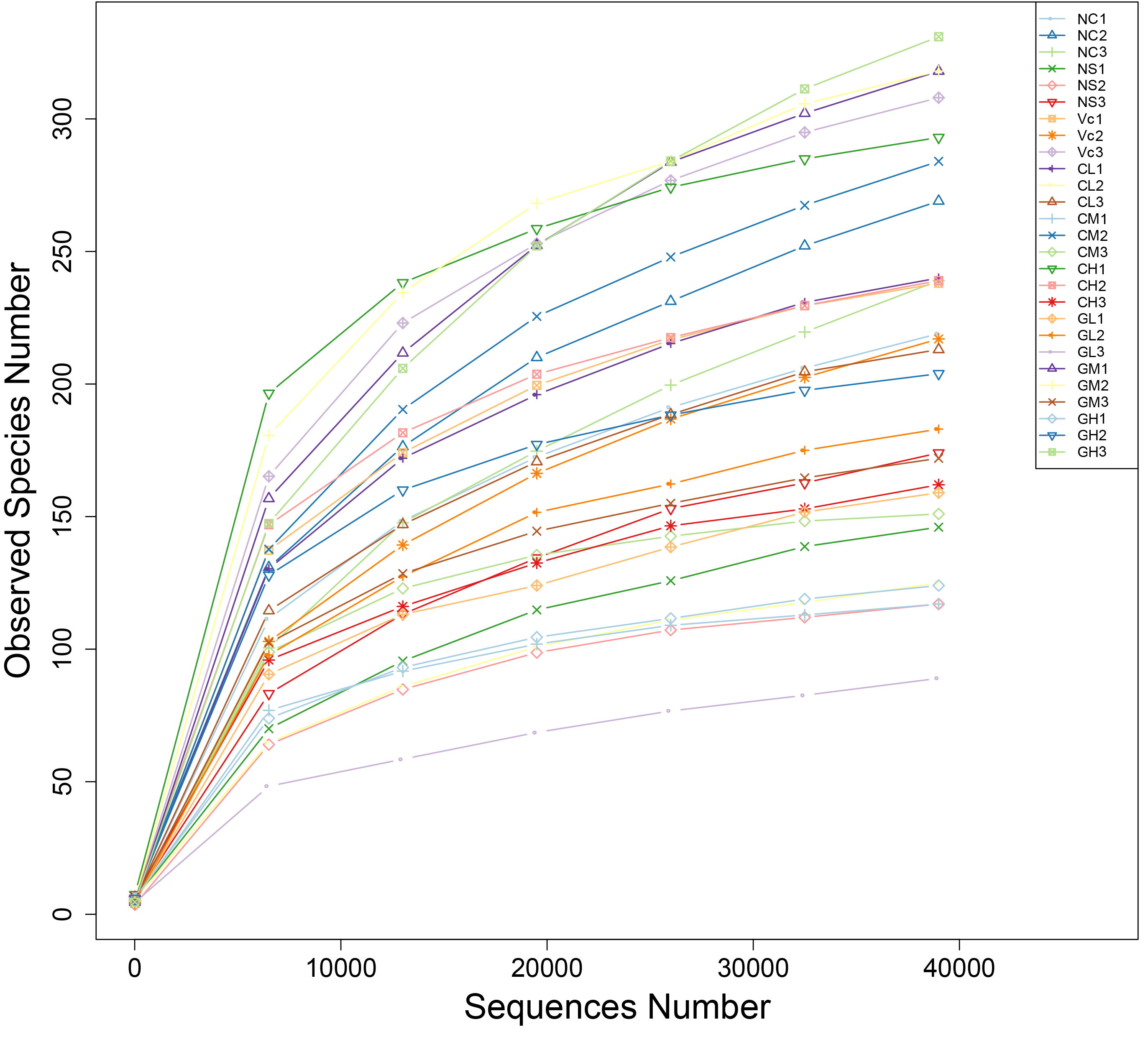

Supplement: S1 Fig — (TIF) [file pone.0248329.s001.tif]

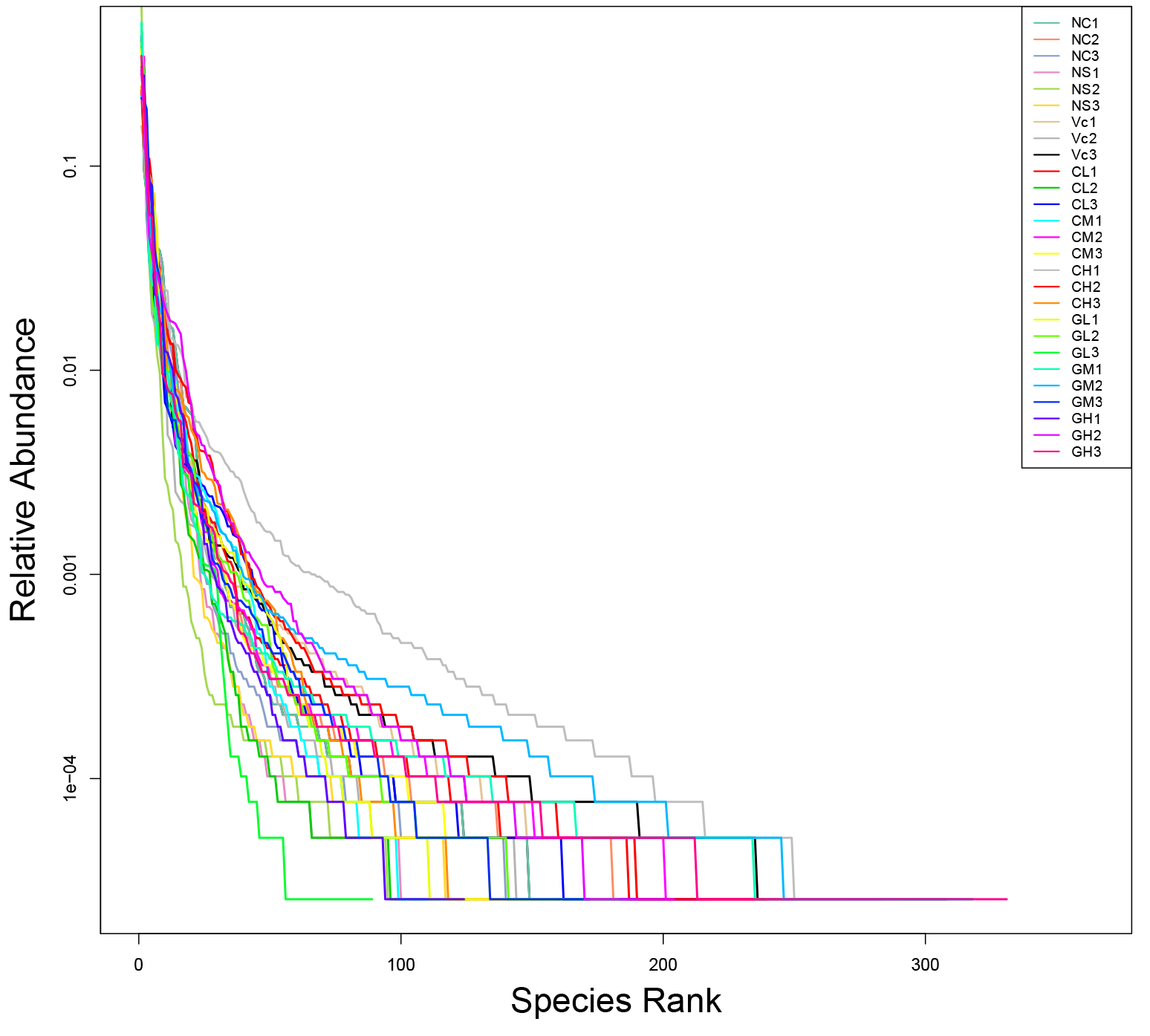

Supplement: S2 Fig — (TIF) [file pone.0248329.s002.tif]

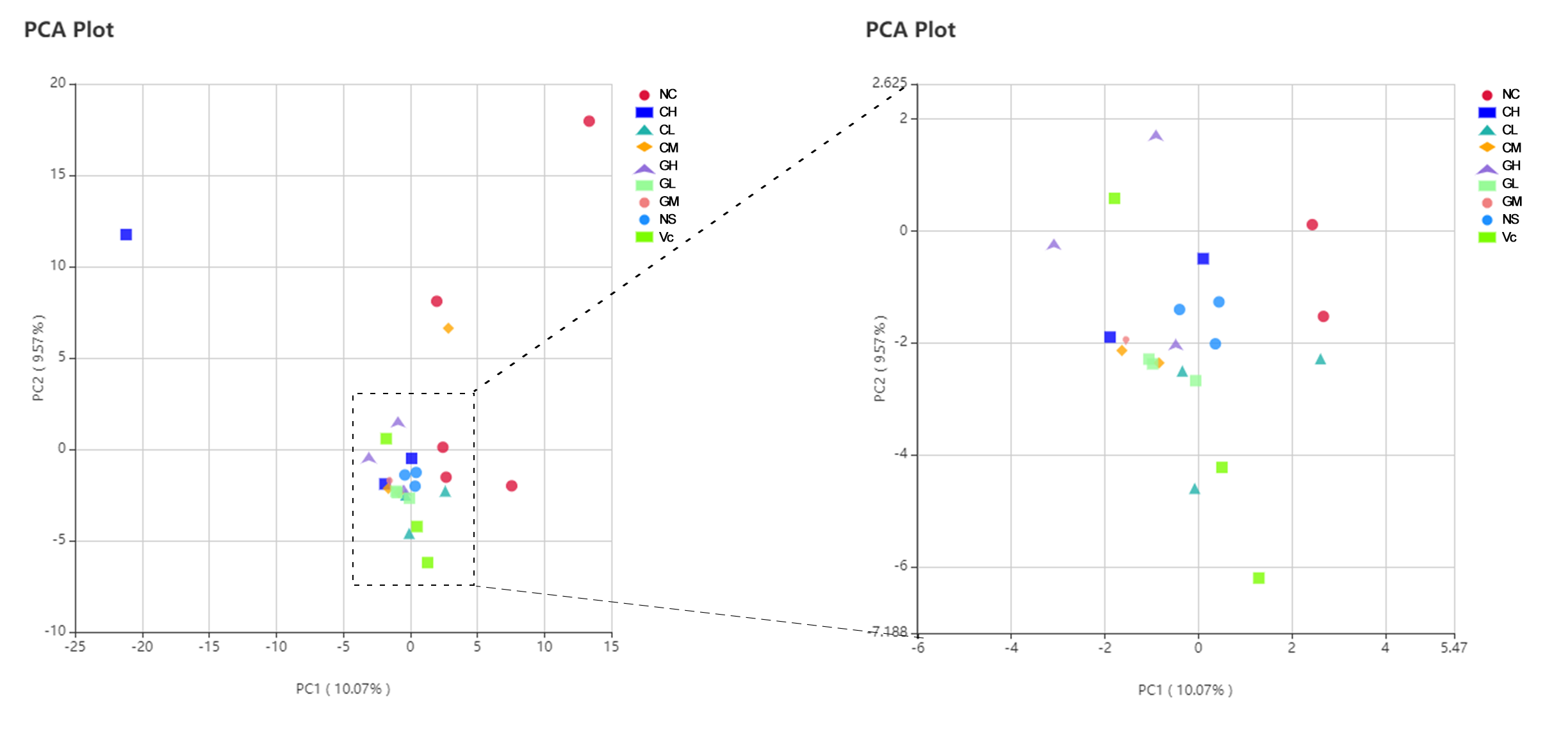

Supplement: S3 Fig — (TIF) [file pone.0248329.s003.tif]
